# Incidence and predictors of regimen-modification from first-line antiretroviral therapy in Thailand: a cohort study

**DOI:** 10.1186/s12879-014-0565-5

**Published:** 2014-10-30

**Authors:** Naho Tsuchiya, Panita Pathipvanich, Nuanjun Wichukchinda, Archawin Rojanawiwat, Wattana Auwanit, Koya Ariyoshi, Pathom Sawanpanyalert

**Affiliations:** Department of Clinical Medicine, Institute of Tropical Medicine, Nagasaki University, 1-12-4, Sakamoto, Nagasaki, 852-8523 Japan; Global COE program, Nagasaki University, 1-12-4, Sakamoto, Nagasaki, 852-8523 Japan; Day Care Center, Lampang Hospital, 280 Paholyothin Road, Muang Lampang, 52000 Lampang, Thailand; Ministry of Public Health, National Institute of Health, 88/7 Tiwanon road, Ampur Muang, 11000 Nonthaburi, Thailand; Food and Drug Administration, Ministry of Public Health, 88/7 Tiwanon road, Ampur Muang, 11000 Nonthaburi, Thailand

**Keywords:** Antiretroviral therapy, Regimen modification, Predictor, Thailand, Resource limited settings, Adverse effects

## Abstract

**Background:**

Antiretroviral therapy markedly reduced mortality in HIV-infected individuals. However, in the previous studies, up to 50% of patients are compelled to modify their regimen in middle and low-income countries where salvage drug is still limited. This cohort study aimed to investigate the incidence and predictors of regimen modification from the first-line antiretroviral regimen in northern Thailand.

**Methods:**

All HIV-infected patients starting antiretroviral therapy (ART) with generic drug (GPOvir®; stavudine, lamivudine and nevirapine) at a governmental hospital in northern Thailand from 2002 to 2007 were recruited. Baseline characteristics and detailed information of regimen modification until the end of 2010 were ascertained from cohort database and medical charts. As a potential genetic predictor of regimen modification, HLA B allele was determined by bead-based array hybridization (WAKFlow® HLA typing kit). We investigated predictors of the regimen modification using Cox’s proportional hazard models.

**Results:**

Of 979 patients, 914 were eligible for the analysis. The observed events of regimen modification was 377, corresponding to an incidence 13.8/100 person-year-observation (95% CI:12.5-15.3) over 2,728 person years (PY) follow up. The main reasons for regimen modification were adverse effects (73.5%), especially lipodystrophy (63.2%) followed by rash (17.7%). Sixty three patients (17.1%) changed the regimen due to treatment failure. 2% and 19% of patients had HLA-B*35:05 and B*4001, respectively. HLA-B*35:05 was independently associated with rash-related regimen modification (aHR 7.73, 95% CI:3.16-18.9) while female gender was associated with lipodystrophy (aHR 2.11, 95% CI:1.51-2.95). Female gender (aHR 0.54, 95% CI: 0.30-0.96), elder age (aHR 0.56, 95% CI: 0.32-0.99) and having HLA-B*40:01 (aHR 0.29, 95% CI: 0.10-0.82) were protective for treatment failure related modification.

**Conclusion:**

HLA-B*35:05 and female gender were strong predictors of regimen modification due to rash and lipodystrophy, respectively. Female gender, elder age, and having HLA-B*40:01 had protective effects on treatment failure-related regimen modification. This study provides further information of regimen modification for future tailored ART in Asia.

**Electronic supplementary material:**

The online version of this article (doi:10.1186/s12879-014-0565-5) contains supplementary material, which is available to authorized users.

## Background

Antiretroviral therapy (ART) has markedly reduced AIDS-related mortality and morbidity [1]. The combination of stavudine (d4T), lamivudine (3TC) and nevirapine (NVP) has been one of the most common first-line ART regimens in resource limited settings. Even after stavudine for newly treated patients is being phased out according to WHO's recommendation, this combination is still widely used in these settings. In Thailand, the fixed-dose generic combination antiretroviral drug, GPOvir® (d4T,3TC and NVP), has been universally available since 2002 [2]. The efficacy of GPOvir® and survival benefit has been demonstrated [2],[3]. Despite of the increased access to ART, adverse effects compelled patients to discontinue or modify the regimen more frequently than ART treatment failure. Previous studies showed that up to 50% of patients modified their first-line regimen due to adverse effects [4]-[9]. A multicenter study in Asia demonstrated that the rate of regimen modification from the combination of d4T/3TC/NVP until October 2004 was 22.3/100 person-year-observation (PYO) and 62.6% were due to adverse effects among patients in Asian and Pacific countries [5]. Major adverse effect resulted in the regimen modification was lipodystrophy, followed by hepatotoxicity and rash in the study. Other studies in resource-limited settings showed the consistent results [6]-[8].

Several demographic, clinical and genetic factors associated with adverse effects have been identified. Female with a higher CD4 cell count, better clinical status and a higher BMI at ART initiation were associated with NVP-induced rash in Thai patients [10]. Another study showed that hepatitis co-infection and elevated alanine aminotransferase increased the risk of hepatotoxicity due to NVP containing ART [11]. In terms of pharmacogenetic factors, HLA-B*57:01 is known to be a strong predictor of hypersensitivity to abacavir [12] and screening has been widely implemented in western countries. In Asia, where the frequency of B*57:01 was low, associations between HLA-B*40:01 and lipodystrophy, and HLA-B*35:05 and rash were reported in small case-control studies [13],[14]. Regimen modification due to these adverse-effects with/without these specific alleles was not evaluated as an outcome in their study.

To our knowledge, only few observational studies comprehensively investigated predictors of ART regimen modification in clinical settings in low- and middle-income countries [6],[7],[15]. This study, therefore, aimed to examine the more recent incidence and predictors of regimen modification of the first-line ART in northern Thailand.

## Methods

### Study site and population

We conducted this study as a sub-study of our previously conducted observational cohort of HIV-infected patients at the HIV centre of a government referral hospital situated in the centre of Lampang province in northern Thailand. Detailed information on the original cohort is presented elsewhere [16]. Participants were all adult (aged >18 years) HIV-infected individuals attending the HIV clinic with given written consent. Any ART-naïve participants in the original cohort who initiated ART with GPOvir® (d4T 30 mg + 3TC 150 mg + NVP 200 mg) until 31 December 2007 were incorporated into this ART patient cohort. Those who experienced ART other than prevention for mother to child transmission (PMTCT) prior to first visit at the HIV centre were excluded. All participants were requested to visit the clinic at least once every 3 months and were followed up until the end of 2010.

### Data collection

The baseline was the time of initiating antiretroviral therapy. The outcome of interest was the first regimen modification from the first-line antiretroviral therapy for any reasons. In this study, regimen modification was defined as any changes in antiretroviral drugs (including single substitutions). Demographic and clinical data at the baseline were obtained from medical records and face-to-face interviews based upon a structured questionnaire. Regimen modification date, one main reason for the regimen modification, and the regimen after modification were ascertained by the cohort database or medical charts. Decision of regimen modification and diagnosis of each adverse effect were made based on the national and international guidelines throughout the observation period. Reasons for regimen modification were coded as follows: adverse effects, treatment failure, drug resistance, pregnancy and treatment for tuberculosis and others. Covariates of interest were: gender, age, mode of HIV transmission, clinical status and CD4 cell count at the baseline, and Human leukocyte antigen (HLA) - B allele.

### Human leukocyte antigen (HLA) genotyping

Blood samples for genotyping were obtained at the baseline. Genomic DNA was extracted from buffy coat using the QIAmp DNA blood Mini Kit (Qiagen, Hilden, Germany) and 4-digit HLA class I typing for B loci was undertaken by bead-based array hybridization (WAKFlow® HLA typing kit, Wakunaga Pharmaceutical Co., Ltd., Hiroshima, Japan) according to manufacturer’s instructions at a commercial laboratory (Kyoto HLA Laboratory, Kyoto, Japan).

### Decision of regimen modification

Throughout the observation period, we made a decision of regimen modification following the WHO guideline which was issued in 2002 [17]. According to the criteria, adverse effect-related regimen modification was considered when; rash with systemic symptoms such as fever, severe rash with mucosal lesions of urticaria, or Stevens-Johnson syndrome or toxic epidermal necrolysis, severe neuropathy, more than 5 times increase of the normal limit in liver enzyme, any type of lactic acidosis. In this criteria, regimen modification due to lipodystrophy was not recommended because of the limited salvage regimen. In the Thai national guideline issued in 2008 and the modified one issued in 2010, it was stated that "Early switching of causative ARV may avoid irreversible lipodystrophy.", but the timing of regimen modification was not clearly defined [18],[19]. The Thai national guideline issued in 2008 which was used during the study period defined criteria for virological failure as HIV viral load(VL) >1000 copies/ml after 6 months of receiving ART with good adherence, or a rebound of VL to >1000 copies/ml in any duration after achieving VL <50 copies/ml [18]. Criteria for immunological failure include: CD4 + T-cell count increases <50 cells/mm 3 after a year of ART; absolute CD4 + T-cell count decreases >30% or percent CD4 decreases >3% from the highest level previously gained; CD4 + T-cell count decreases to the level lower than pre-ART level [18]. Clinical failure is defined as clinical relapse of prior OI or occurrence of a new OI [18]. However, practically, annual VL testing has not been fully implemented until late 2000's. Thus our definition of treatment failure was mainly by immunological and clinical failure in this study. Immunological failure and clinical failure are given less importance in the current national guideline because of low sensitivity and specificity, but still used in other resource-limited countries [19].

This study was approved by the Thai Government Ethics Committee in December 1999 and December 2005.

### Statistical analysis

The primary endpoint was the time to the first regimen modification after starting ART with GPOvir®. All patients were censored at the last visit date before data collection if no event of regimen modification or death had occurred; the last censored date was 31 December 2010. In case of death, moving, and changing to other hospitals, the data were censored at that time point as well. After descriptive summaries, we calculated incidence rates of regimen modification with 95% confidence intervals (CI). Kaplan-Meier estimates for the probability of survival without regimen modification was performed. After identifying the reasons for regimen modification, Cox's proportional hazard models were fitted in order to identify the factors associated with regimen modification for different reasons. In the multivariable models, we put variables with p-value under 0.05 in the univariable analysis and covariates of interest. All statistical analyses were performed using STATA version 12.0 (Stata corp, College station, Texas).

## Results

### Baseline characteristics of study participants

**Figure 1:**
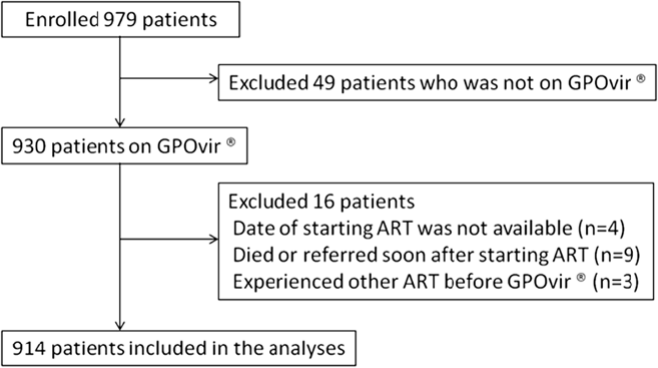
**Recruitment of patients.** ART, antiretroviral therapy.

Between 1 August 2002 and 31 December 2007, 979 ART-naive HIV-infected patients started ART with GPOvir® at the HIV clinic in Lampang hospital. Of these, 49 patients who actually did not start GPOvir® and 16 patients who did not meet the criteria were excluded. Subsequently, 914 participants were included in the analyses (Figure 1). Table 1 shows baseline characteristics at the initiation of ART and frequency of HLA-B*35:05 and B*40:01. Almost half of the participants were male and the majority of patients got infected with HIV via heterosexual transmission. At the initiation of ART, around 60% of patients had already had AIDS defined illness and the median CD4 cell count was as low as 50 cells/ml (Interquartile range, IQR 17-118). Out of 908 participants whose data on HLA-B allele was available, 18 (2.0%) had HLA-B*35:05 allele and 168 (18.5%) had B*40:01 allele. Frequency of other HLA alleles was shown in Additional file 1: Table S1.

**Table 1:** **Baseline characteristics of patients included in the analysis (N = 914)**

Gender	
Male (Number, %)	461 (50.4)
Age (Median, IQR^a^)	34.6 (30.6-39.6)
Mode of transmission (Number, %)	
Heterosexual	848 (92.8)
Homosexual	15 (1.6)
IDU^b^	29 (3.2)
Others/unknown	22 (2.4)
Clinical symptoms (Number, %)	
Asymptomatic	124 (13.6)
Symptomatic	217 (23.7)
AIDS	549 (60.1)
Unknown	24 (2.6)
CD4 cell count (cells/*μ*l, median, IQR^a^)	50 (17-118)
Carrying HLA B*35:05 (Number, %)	18 (2.0)
Carrying HLA B*40:01 (Number, %)	168 (18.5)

### Regimen modifications and reasons

The median follow up from ART initiation was 89.8 months. The observed events of regimen modification were 377 over 2,728 person years (PY) of follow up, corresponding to the incidence rate of 13.8/100PYO (95% CI 12.5-15.3).

**Figure 2:**
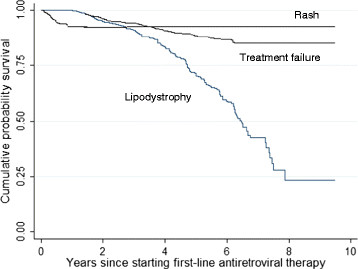
**Kaplan-Meier curve of regimen modification stratified by reasons.**

Table 2 summarizes the reasons for the regimen modification and time from ART initiation. The most common reason for modification was adverse effects of GPOvir® (73.5%), followed by treatment failure (16.7%), for treatment of tuberculosis (TB) (4.2%), and pregnancy (2.7%). For the patients who developed TB or became pregnant, GPOvir® was changed to the combination of d4T/3TC/Efavirenz(EFV) and zidovudine/3TC/NVP, respectively. Detailed distribution of adverse effects resulting in regimen modification is also shown with days to regimen modification. Lipodystrophy (63.2%), rash (17.7%), and hepato-toxicity (11.9%) were the most represented adverse effects. Peripheral neuropathy was not the main reason for regimen modification (data not shown). The median days to regimen modification due to lipodystrophy was longest while rash-related regimen modification occurred earliest. Figure 2 shows the Kaplan-Meier estimates of regimen modification by reasons for the modification; lipodystrophy, rash and treatment failure. Lipodystrophy-related regimen modification was detected almost throughout the observation period, but rash-related regimen modification was seen only in the first one year.

**Table 2:** **Reasons for regimen modification (n = 377)**

Reasons	Number (%)	Days to regimen modification
		(Median, IQR^a^)
Adverse effects	277 (73.5)	29 (5-55)
Lipodystrophy	175 (63.2)	1493 (936-1946)
Rash	49 (17.7)	29 (24-44)
Hepatitis/Liver dysfunction	33 (11.9)	146 (56-315)
Lactic acidosis	9 (3.3)	511 (410-573)
Neuropathy	8 (2.9)	506 (286-574)
Others	3 (1.1)	545 (388-1769)
Treatment failure	63 (16.7)	1147 (344-1246)
For TB^b^ treatment	16 (4.2)	186 (93-248)
Pregnancy	10 (2.7)	868 (682-1643)
Others	2 (0.5)	527 (62-992)
Unknown	9 (2.4)	1085 (527-1705)

^a^IQR, interquartile range.

^b^TB, tuberculosis.

### Predictors of regimen modification

As we assumed that predictors were different depending on reasons for regimen modification, we fitted Cox's proportional hazard models to elucidate　the factors associated with reason-specific regimen modification. Covariates included in multivariable models were: gender, age, clinical status, CD4 cell count at the baseline and selected HLA-B allele. Factors independently associated with regimen modification due to lipodystrophy was being female [adjusted hazard ratio (aHR) 2.11, 95% confidence interval (95% CI) 1.51-2.95, p-value < 0.001]. Age of over 35 was also weakly associated with lipodystrophy-related regimen modification (aHR 1.35, 95% CI 0.98-1.84, p-value = 0.06) whereas there was no association between HLA-B*40:01 (Table 3) and lipodystrophy-related modification. Females had a higher hazard of rash-related regimen modification (aHR 1.82, 95% CI 0.99-3.34, p-value = 0.05). Intriguingly HLA-B*35:05 was by far the strongest risk of regimen modification due to rash. Among 890 patients who do not have HLA-B*35:05, forty two (4.7%) modified the regimen due to rash, whereas 6 (33.3%) of 18 patients with HLA-B*35:05 developed a rash resulting in the regimen modification corresponding to aHR of 7.73 (95% CI 3.16-18.9, p-value < 0.001) (Table 4). CD4 cell count and clinical status at the initiation of ART did not show any association with regimen modification due to these adverse effects. None of demographic, clinical and genetic factors were associated with regimen modification due to hepatotoxicity (data not shown). Female (aHR 0.54, 95% CI 0.30-0.96, p-value = 0.04) and elder age (aHR 0.56, 95% CI 0.32-0.99, p-value = 0.05) were associated with a decreased hazard of treatment failure-related regimen modification. Interestingly, the association with HLA B*40:01 remained significant after adjusting other factors; those who has HLA-B*40:01 showed over 70% lower hazard of treatment failure-related modification than that of those without HLA-B*40:01 (aHR 0.29, 95% CI 0.10-0.82, p-value = 0.02) (Table 5).

**Table 3:** **Predictors of regimen modification due to lipodystrophy**

	HR^a^(95% CI^b^)	*P*	aHR^c^(95% CI^b^)	*P*
Gender				
Male	1		1	
Female	2.05 (1.50-2.79)	<0.001	2.11 (1.51-2.95)	<0.001
Age				
<35	1		1	
≥35	1.12 (0.83-1.51)	0.46	1.35 (0.98-1.84)	0.06
Clinical status				
Asymptomatic	1		1	
Symptomatic	0.88 (0.54-1.45)	0.62	0.81 (0.48-1.36)	0.43
AIDS	0.65 (0.41-1.03)	0.07	0.72 (0.43-1.21)	0.21
CD4 cell count				
>200	1		1	
50-199	0.85 (0.46-1.57)	0.61	0.95 (0.50-1.80)	0.87
<50	0.80 (0.43-1.46)	0.46	1.08 (0.56-2.07)	0.83
HLA-B*40:01				
No	1		1	
Yes	0.92 (0.64-1.32)	0.64	0.91 (0.62-1.32)	0.62

^a^HR, hazard ratio.

^b^CI, confidence interval.

^c^aHR, adjusted hazard ratio.

**Table 4:** **Predictors of regimen modification due to rash**

	HR^a^(95% CI^b^)	*P*	aHR^c^(95% CI^b^)	*P*
Gender				
Male	1		1	
Female	1.83 (1.03-3.27)	0.04	1.82 (0.99-3.34)	0.05
Age				
<35	1		1	
≥35	1.20 (0.69-2.10)	0.51	1.08 (0.60-1.93)	0.81
Clinical status				
Asymptomatic	1		1	
Symptomatic	0.45 (0.19-1.09)	0.08	0.52 (0.21-1.28)	0.15
AIDS	0.61 (0.31-1.22)	0.16	0.94 (0.44-2.03)	0.88
CD4 cell count				
>200	1	0.75	1	0.60
50-199	1.21 (0.37-3.98)		1.38 (0.41-4.63)	
<50	0.70 (0.21-2.39)	0.58	0.71 (0.20-2.55)	0.60
HLA-B*35:05				
No	1		1	
Yes	8.24 (3.51-19.4)	<0.001	7.73 (3.16-18.9)	<0.001
HLA-B*57:01				
No	1		1	
Yes	1.70 (0.23-12.3)	0.60	2.06 (0.28-15.4)	0.48

^a^HR, hazard ratio.

^b^CI, confidence interval.

^c^aHR, adjusted hazard ratio.

**Table 5:** **Predictors of regimen modification due to treatment failure**

	HR^a^(95% CI^b^)	*P*	aHR^c^(95% CI^b^)	*P*
Gender				
Male	1		1	
Female	0.61 (0.36-1.01)	0.06	0.54 (0.30-0.96)	0.04
Age	1		1	
<35				
≥35	0.54 (0.32-0.92)	0.02	0.56 (0.32-0.99)	0.05
Clinical status				
Asymptomatic	1		1	
Symptomatic	1.29 (0.51-3.23)	0.59	1.19 (0.47-3.00)	0.72
AIDS	0.92 (0.39-2.20)	0.86	0.73 (0.29-1.85)	0.51
CD4 cell count				
>200	1		1	
50-199	0.74 (0.28-1.94)	0.55	0.59 (0.22-1.55)	0.28
<50	0.82 (0.32-1.12)	0.68	0.68 (0.25-1.86)	0.45
HLA-B*40:01				
No	1		1	
Yes	0.38 (0.16-0.87)	0.02	0.29 (0.10-0.82)	0.02

^a^HR, hazard ratio.

^b^CI, confidence interval.

^c^aHR, adjusted hazard ratio.

## Discussion

Since the choices of salvage regimen are still limited in most of low- and middle-income countries, well-managed first line ART is essential. Repeated investigation of the incidence of regimen modification and its determinants will help to keep patients on the first ART regimen as long as possible.

In the current study, 377 (41.4%) of HIV-infected patients experienced regimen modification from the first line regimen using GPOvir® over the observation period. This proportion is similar to that reported from previous studies [5],[7],[8],[15]. The incidence rate of regimen modification of 13.8/100 PYO (95% CI:12.5-15.3) was lower than that of 22.3/100 PYO reported in the previously published multicentre study in Southeast Asia [5]. This difference might reflect the differences in the availability of salvage regimen and definition of regimen change among countries. Consistent with other studies, adverse effects, especially lipodystrophy and rash, were the represented reasons for the ART regimen modification in the present study [5],[7],[20],[21]. Frequency and distribution of adverse effects as the reasons for the regimen modification were almost similar to those in other studies in Asia [5],[22].

Predictors of the specific cause of regimen modification varied. From the risk factor analysis for each cause of regimen modification, female patients had two times higher risk of regimen modification due to lipodystrophy. Some studies reported that female gender was a risk factor for development of lipodystrophy [23],[24], but it is difficult to know whether the regimen change due to lipodystrophy was related to the gender difference in this biological mechanism or simply because female were more conscious about the change in body composition for cosmetic reasons. Similar to previous studies, older age was also increased the risk of lipodystrophy-related regimen modification [23],[24]. In a small case-control study in Thailand, Wangsomboonsiri *et al.* found the strong association between HLA-B*40:01 and development of stavudine-associated lipodystrophy [13]. Their definition of lipodystrophy was based on the strict clinical measurements, whereas we focused on the documented lipodystrophy which was severe enough to induce regimen modification. No association in our study might be due to differences in the definition of outcome.

This is the first study demonstrating that HLA-B*35:05 strongly associated with rash-related regimen modification in a longitudinal observation. A small case-control study reported that HLA-B*35:05 allele was observed in 18% of patients with NVP-induced rash and only in 1% of those who were NVP tolerant in Thailand. They presented an increased risk of rash among those who had HLA-B*35:05 (aOR 49.2, 95% CI 6.5-374.4, p-value = 0.0002) [14]. In our study, the frequency of HLA-B*35:05 among those who experienced rash-related regimen modification was slightly lower than that in previous study investigated all rash regardless of regimen modification. This discrepancy could be explained by the difference in the outcome measurement. Also, the lead-in dosing of NVP for all our participants might have played some role to reduce the risk of NVP-related rash. Unlike a single HLA class I allele of B*57:01 related abacavir hypersensitivity, different class I and class II associations have been found with NVP rash, hypersensitivity, and hepatitis [25]. Regional differences in class I HLA association are noted [26]-[30], partly because of the difference in HLA allele frequency distribution. As observed association between HLA-B*35:05 and regimen modification due to rash was strong, we believe that screening HLA-B*35:05 will reduce rash-induced regimen modification and related cost, especially in the region where the frequency of HLA-B*35:05 is high.

Regimen modification due to treatment failure has a different mechanism. One of the most important factors to be considered is adherence. We did not collect this kind of information since it was out of the scope of this study. However, our result that male and younger age as risk factors of regimen modification due to treatment failure is consistent with the results of our previous study with respect to adherence, treatment failure and its predictors [31]. Of note, our study showed the protective effect of HLA-B*40:01 on regimen modification due to treatment failure. Accumulating data of HLA class I alleles has been described as affecting the evolution of the HIV-sequence on both individual and population levels [32]-[36]. HLA-driven immune pressure may delay development of critical drug resistance mutations and lead to a better treatment outcome in some patients. To date HLA-B*40:01-associated mutation has not been described as drug resistance mutations. Although lack of information with respect to mutation and other qualitative data, our results might explain the association between HLA-B*40:01 and treatment efficacy. Another point to be carefully considered is that the incidence of treatment failure-related regimen modification might be underestimated in our study. Since we focused on the first regimen modification, those who experienced side effects-related modification before treatment failure were not included in the population at risk of treatment failure-related modification.

In this study, time to regimen modification due to lipodystrophy was around three times longer than other studies [5],[6]. It might reflect the time lag between the diagnosis of lipodystrophy and regimen modification. Compared with other adverse effects and treatment failure, the timing of lipodystrophy-related regimen modification was decided based on more subjective reasons such as patients' preference and perception toward the appearance change rather than clinicians' decision. In our experience, some patients are reluctant to change the regimen in fear of new adverse effects or less efficacy. Among patients using the regimen of d4T/3TC/NVP, lipodystrophy is mainly due to d4T. Since around 2008, according to the strong recommendation from WHO, zidovudine has been substituted for d4T. In Thailand, national guideline was revised and generic combination tablet of AZT/3TC/NVP (GPO-Z®) has been recommended as the main first-line ARV since October 2008 [18]. However, as the combination of d4T/3TC/NVP is still one of the most commonly used first-line regimen in resource limited settings, our findings will be beneficial for clinicians in these settings to identify patients at the risk of regimen modification.

Our study has several limitations. Firstly, we could not identify the specific drug which was responsible for B*35:05 associated rash, though previous data indicates that NVP is most likely. Secondly, regimen modification may have been underestimated, especially because regimen modification due to lipodystrophy took longer time than other studies. However, we believe that our data reflects more the real situation in the government sector. Thirdly, we do not have detailed clinical information at the initiation of ART such as body mass index, haemoglobin level and viral load, since this study was prospective observational study and the data collection was not specifically designed for identifying the factors associating with regimen modification. Some studies reported that these factors increased the risk of early mortality [37] (maybe without regimen modification) and some adverse effects [8]. It also may lead to underestimation of regimen modification.

## Conclusions

In conclusion, we present a moderate incidence rate of regimen modification from a clinical setting in northern Thailand. Adverse effects were the main reasons for regimen modification. Female gender and older age appeared to be risk factors of regimen modification due to lipodystrophy. Individuals with HLA-B*35:05 more likely to experience rash-related regimen modification. Male and younger age increased the risk of treatment failure-related regimen modification, while female gender, older age and having HLA-B*40:01 showed protective effects.

Our findings have several implications for the better management of HIV-infected individuals on ART. Screening for HLA-B*35:05 will reduce the risk of rash-related regimen modification while HLA-B*4001 could be a predictor for durability of the combination of d4T/3TC/NVP. Finally, to reduce lipodystrophy-related regimen modification, accelerated implementation of WHO recommendation for phasing-off d4T will be crucial.

## Additional file

## Electronic supplementary material

Additional file 1: Table S1.: Frequency of HLA-B allele (n=908). (DOCX 14 KB)

Below are the links to the authors’ original submitted files for images.

Authors’ original file for figure 1

Authors’ original file for figure 2
