# Supplementary material for: Incidence and predictors of regimen-modification from first-line antiretroviral therapy in Thailand: a cohort study
Source: BMC Infect Dis. 2014 Oct 30;14:565. doi: 10.1186/s12879-014-0565-5 (PMC4226857; doi:10.1186/s12879-014-0565-5)
Supplement: Supplementary file 1 — Additional file 1: Table S1.: Frequency of HLA-B allele (n=908). (DOCX 14 KB) [file 12879_2014_565_MOESM1_ESM.docx]

Table S1. Frequency of HLA-B allele (n=908)

| Allele | Number (%) |
| --- | --- |
| B*0705 | 33 (3.6) |
| B*0801 | 11 (1.2) |
| B*1301 | 158 (17.4) |
| B*1302  B*1501  B*1502  B*1507  B*1512  B*1525  B*1527  B*1801  B*1802  B*2704  B*2706  B*3501  B*3503  Allele  B*3505  B*3802  B*3901  B*4002  B*4006  B*4403  B*4601  B*5101  B*5102  B*5201  B*5502  B*5601  B*5701  B*5801 | 17 (1.9)  27 (3.0)  120 (13.2)  18 (1.2)  10 (1.1)  50 (5.5)  9 (1.0)  25 (2.8)  17 (1.9)  41 (4.5)  35 (3.9)  14 (1.5)  11 (1.2)  Number (%)  18 (2.0)  87 (9.6)  14 (1.5)  27 (3.0)  23 (2.5)  43 (4.7)  267 (29.4)  84 (9.3)  29 (3.2)  63 (6.9)  46 (5.1)  22 (2.4)  11 (1.2)  134 (14.8) |
