# Supplementary figures and images for: Incidence and predictors of regimen-modification from first-line antiretroviral therapy in Thailand: a cohort study
Source: BMC Infect Dis. 2014 Oct 30;14:565. doi: 10.1186/s12879-014-0565-5 (PMC4226857; doi:10.1186/s12879-014-0565-5)

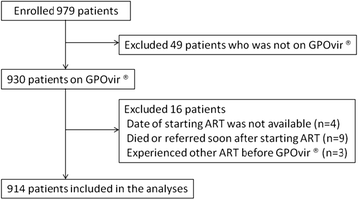

Supplement: Supplementary file 2 — Authors’ original file for figure 1 [file 12879_2014_565_MOESM2_ESM.gif]

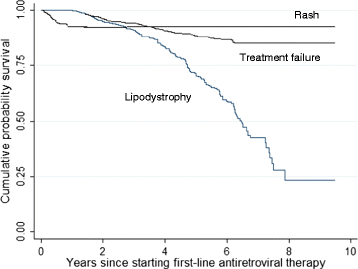

Supplement: Supplementary file 3 — Authors’ original file for figure 2 [file 12879_2014_565_MOESM3_ESM.gif]
